# Assessment of antimicrobial, cytotoxicity, and antiviral impact of a green zinc oxide/activated carbon nanocomposite

**DOI:** 10.1038/s41598-022-12648-w

**Published:** 2022-05-24

**Authors:** Hassan S. Hassan, Deyaa Abol-Fotouh, Eslam Salama, Marwa F. Elkady

**Affiliations:** 1 Environmental Engineering Department, Egypt-Japan University of Science and Technology (E-JUST), New Borg El-Arab City, Alexandria 21934 Egypt; 2 Electronic Materials Researches Department, Advanced Technology and New Materials Research Institute (ATNMRI), City of Scientific Research and Technological Applications (SRTA-City), New Borg El-Arab City, P.O. Box 21934, Alexandria Egypt; 3 Environment and Natural Materials Research Institute (ENMRI), City of Scientific Research and Technological Applications (SRTA-City), New Borg El-Arab City, Alexandria 21934 Egypt; 4 Chemical and Petrochemical Engineering Department, Egypt-Japan University of Science and Technology (E-JUST), New Borg El-Arab City, Alexandria 21934 Egypt; 5 Fabrication Technology Department, Advanced Technology and New Materials Research Institute (ATNMRI), City of Scientific Research and Technological Applications (SRTA-City), New Borg El-Arab City, Alexandria 21934 Egypt

**Keywords:** Microbiology, Nanoscience and technology

## Abstract

This work deals with the synthesis of zinc oxide nanoparticles/activated carbon (ZnO NPs/AC) nanocomposites with different weight ratios (3:1, 1:1, and 1:3), where the antimicrobial, antiviral, and cytotoxicity impact of the formulated nanocomposites were evaluated versus the crude ZnO and AC samples. The formula (3:1; designated Z3C1) exhibited the utmost bactericidal effect against Gram positive group, unicellular and filamentous fungi. Regarding Gram negative group, the sample (Z3C1) was remarkably effective against *Klebsiella pneumonia*, unlike the case of *Escherichia coli*. Moreover, the whole samples showed negligible cytotoxicity against the human WI38 cell line, where the most brutality (4%) was exerted by 1000 µg/mL of the formula (Z1C3). Whilst, the formula (Z3C1) exerted the apical inhibition impact against *Herpes simplex* (HSV1) virus. Consequently, the synthesized (Z3C1) nanocomposite was sorted out to be fully characterized via different physicochemical techniques including FTIR, XRD, SEM, TEM, Zeta potential, TGA, and BET. XRD indicated a predominance of the crystalline pattern of ZnO NPs over the amorphous AC, while the FTIR chart confirmed an immense combination between the ZnO NPs and AC. SEM, TEM, and size distribution images illustrated that the fabricated ZnO NPs/AC was in the nanoscale size swung from 30 to 70 nm. The distinctive surface area of composite material, recording 66.27 m^2^/g, clearly disclosed its bioactivity toward different bacterial, fungal, and virus species.

## Introduction

One of the most important public health issues in recent decades has been the emergence of antibiotic-resistant microbial infections. The widespread use of antibiotics, as well as inadequate consumption, are contributing to this issue. Among the most successful strategies for reducing the severity of this crisis is to develop novel and thoroughly effective nano-antimicrobial drugs that tackle multi-drug resistance^1,2^.

Zinc oxide nanoparticles (ZnO NPs) have drawn significant attention for their unique optical and chemical properties^3^. Chemically, the surface of ZnO is rich in -OH groups, which permit ZnO to slowly dissolve in both acidic (e.g., the tumour cells and tumour microenvironment) and strong basic conditions. Besides, their nanosized nature leads to changes in the chemical, mechanical, electrical, structural, morphological and optical properties of the material, which enables nanoparticles to interact uniquely with cellular biomolecules, facilitating the physical exchange of nanoparticles into inner cellular structure^4^.

Therefore, in different biological and clinical applications, the ZnO NPs have been broadly examined as anti-angiogenesis, antiplatelet agents, anti-inflammatory, antioxidant, dental materials, cosmetics, drug and gene delivery agent^5,6^. Moreover, ZnO NPs have tremendous potential in biological applications like biological sensing, biological labelling, and nanomedicine along with their acaricidal, pediculicidal, larvicidal and anti-diabetic activities^7^. Additionally, ZnO NPs have attained an impressive antimicrobial behaviour (antiviral, antibacterial, and antifungal) due to their expanded specific surface area, as the reduced particle size leads to enhanced particle surface reactivity^8–10^.

In comparison to most of the other metal oxide NPs, ZnO NPs are not only cheaper but relatively non-toxic, biocompatible, and environmentally mild as well. Therefore, there has been increased interest in developing green and cost-effective synthesis approaches for ZnO NPs^11,12^.

The water hyacinth (*Pontederia crassipes*, also known as *Eichhornia crassipes*) is one of the world’s most invasive weeds, having a devastating impact on the economy and ecology, particularly in tropical and sub-tropical locations. Extensive efforts have been made not only to reduce the spread of water hyacinth, but also to use the biomass collected to produce extensively valuable products such as biofuels (bioethanol, biohydrogen), biopolymers (cellulose, hemicelluloses), levulinic acid, biofertilizers, and not to mention super adsorbent activated carbon (AC)^13,14^.

Activated carbon (AC) has recently grabbed raised interest due to its impressive characteristics, where it exhibits a prominent adsorption capacity, a well-developed and easy-to-modify surface, notable chemical stability, and mechanical strength. AC is primarily created via pyrolysis and chemical or physical activation of organic precursors at elevated temperatures, where it can be manufactured in huge quantities for use in adsorption, separation, and a variety of other applications. Because of its exceptional ability to adsorb pathogens, spores, and a variety of toxins including bilirubin, toxic metals, and protein-bound uremic toxins, AC has been recommended for the treatment of subcutaneous and gastrointestinal infections over the world^15,16^.

Furthermore, as compared to the results of only the active chemicals, multiple investigations have shown that AC mostly enhances the action of any adsorbed active antiviral or antibacterial drugs^17,18^.

In this study, we reported the synthesis of AC from locally obtained biomass of water hyacinth, and the generated AC was used to propagate a nanocomposite with ZnO NPs in different ratios of both ingredients. A wide assessment of the ZnO NPs/AC formulations' antibacterial, antifungal, and antiviral properties was conducted before testing their cytotoxicity against a human cell line to learn more about their bio-safety. Following that, the most effective ZnO NPs/AC formula was picked and subjected to a thorough characterization plan to determine its physical and chemical properties.

## Results and discussion

### Antimicrobial activity of ZnO NPs/AC nanocomposites

We have adopted the synthesised ZnO NPs and AC to formulate three nanocomposites with different weight ratios, where the ZnO NPs: AC proportions were; 3:1, 1:1, and 1:3 and they were designated as; (Z3C1), (Z1C1), and (Z1C3) in respective order.

The impact of the neat ZnO NPs, neat AC, and the synthesized ZnO NPs/AC nanocomposites on various microorganisms was determined. Generally, it was evident that the sample that included only ZnO NPs exhibited distinctive antimicrobial activities against almost all the test organisms, which is equal to or with greater extent than that of the control broad-spectrum antibiotic.

**Figure 1:**
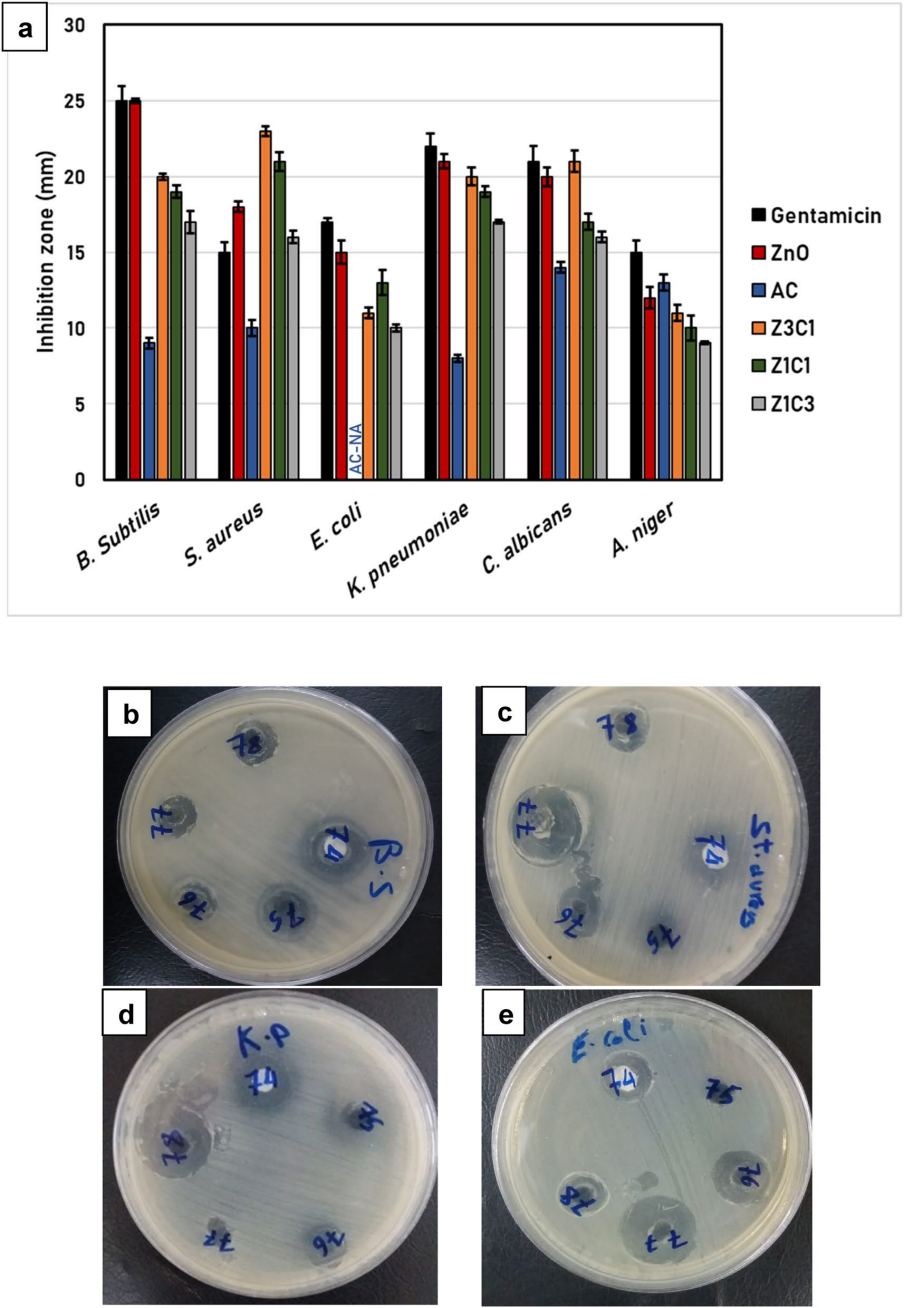
(**a**) Antimicrobial impact of the synthesized ZnO/AC nanocomposites against Gram-positive, Gram-negative bacteria, and unicellular and multicellular fungi. The values are the average of 3 replicates expressing the diameter of the clear zones (mm), while the error bars represent standard deviations. (NA): no activity; Samples for the antimicrobial impact of the synthesized nanocomposites against (**b**) *Bacillus subtilis*; (**c**) *Staphylococcus aureus*; (**d**) *Klebsiella pneumonia*; and (**e**) *Escherichia coli*. The codes of the wells are referring to the following: (74): neat ZnO NPs; (75): neat AC; (76): Z3C1; (77): Z1C1; (78): Z1C3.

Moreover, the formulations of the ZnO NPs/AC found to show superior antimicrobial activity comparing to the neat ZnO NPs, particularly the sample (Z3C1) which recorded the highest impact against the strain *S. aureus* among all examined samples. On the other hand, the tested nanocomposites appeared less effective against the *E. coli* strain, where the neat AC showed no activity at all (Fig. 1a).

Regarding the antifungal effect, the ZnO NPs/AC nanocomposites showed an impact on the yeast *C. Albicans,* where the nanocomposite sample (Z3C1) attained almost the same influence as the neat ZnO NPs and the control antimicrobial agent (Gentamicin). Nevertheless, the tested ZnO NPs/AC nanocomposites exhibited fluctuated influence against the multicellular fungi *Aspergillus niger*, where the loftiest inhibition zone was recorded by neat ZnO NPs and neat AC, which exceeded two-thirds of the effect of the control antimicrobial agent as shown in Fig. 1b–e.

According to Jin and his co-worker^16^, the suggested antimicrobial mechanism owing to the ZnO NPs may be briefly explained in five steps: (1) microbial cells confined and invaded by the ZnO NPs, which results in; (2) generation of reactive oxygen species (ROS), and (3) malfunction of the intracellular energy generating system, which leads to (4) disintegration of the protective cellular membranes, and (5) DNA replication failure and decomposition of the genetic material.

Furthermore, the enhanced antimicrobial properties of the ZnO/AC nanocomposites may be mainly attributed to the probable synergistic effect of the added AC, and the synthesizing route utilized for the preparation ZnO/AC nanocomposite. Although it is suggested the elevated surface area of the activated carbon (AC) material has a crucial role in the growth inhibition of microorganisms, Das et al. reported that other characteristics of the AC, like the particle's sharp edges and the existence of multiple functional groups, probably contribute the antimicrobial impact of the AC^19^.

### Cytotoxicity of the ZnO/AC nanocomposites on the human cell line (WI38)

**Figure 2:**
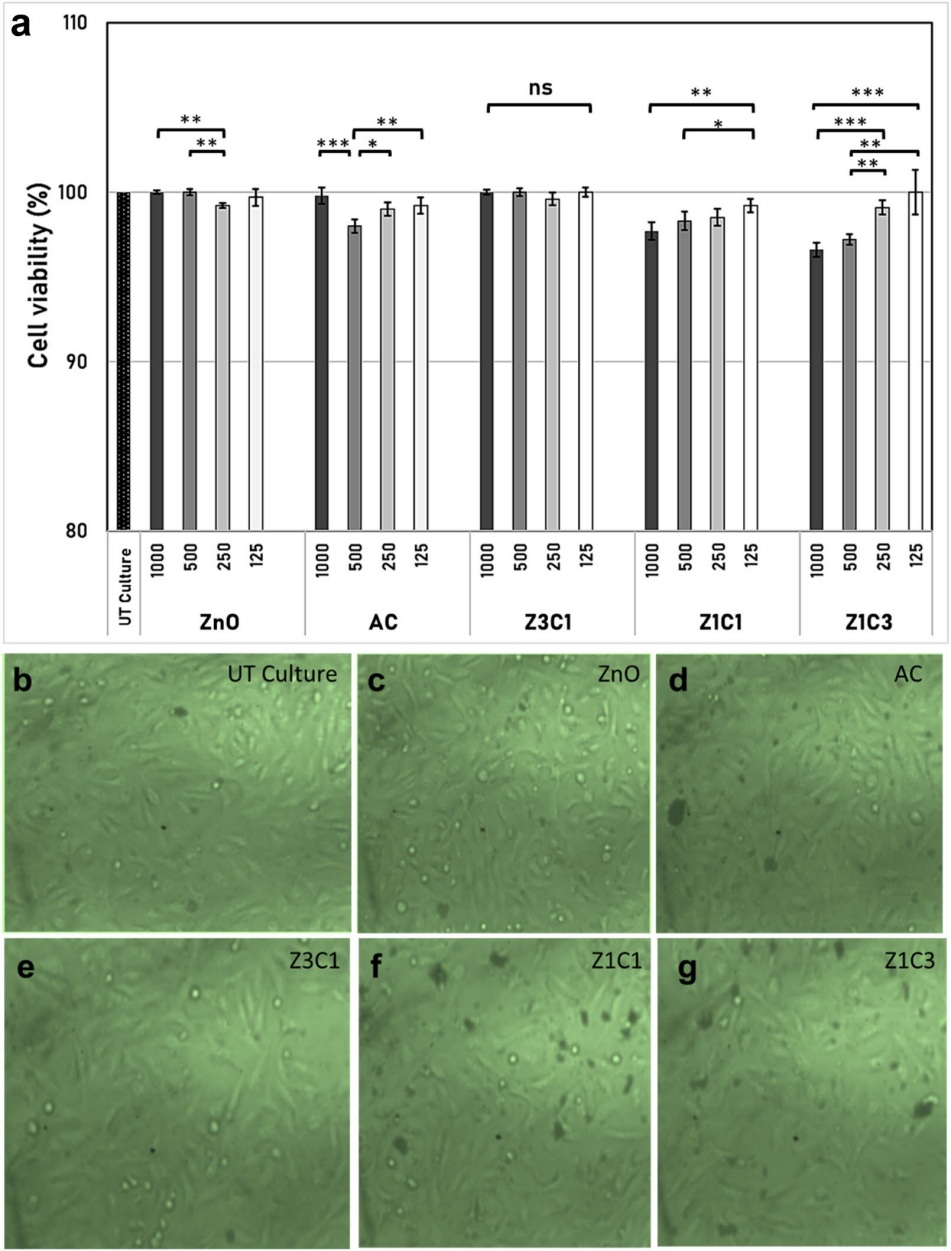
Cytotoxicity of multi concentrations of the synthesized ZnO NPs/AC nanocomposites (µg/mL) on the WI38 human cell line compared to the untreated (UT culture) cell line. The error bars are the estimated standard deviations (n = 3). The (ns) refers to a non-significant difference, where *p* > 0.05. *p < 0.05, **p < 0.01, and ***p < 0.001; (**b**) the microscopic image of the untreated WI38 cell line; (**c**–**g**) cell lines treated with the ZnO NPs/AC composites (1000 µg/mL).

Herein, the neat ZnO NPs, neat AC, and the synthesized ZnO/AC nanocomposites were all tested against the WI38 cell line to inspect their cytotoxicity. Throughout the whole examined samples and at all adopted dilution factors, there were no severe or even mild impacts on the growth of the WI38 cell line, and the maximum toxicity was recorded by the sample (Z1C3) at the 1000 µg/mL concentration to be about 4% inhibition value (Fig. 2a).

It has been reported that the MTT assay is not fully valid for determining cytotoxicity because it may overestimate the active cell number unless the treated cells were inspected by the microscope^20^. Therefore, the microscopic investigation of the untreated control cell line and those treated with the synthesized ZnO/AC nanocomposites revealed the normal morphology of the WI38 cells and the absence of any cellular deformations or anomalies. Moreover, the cell counts appeared to be relevant to the findings of the MTT assay. The microscopic images of the control and the treatments of the upmost concentrations of the examined formulas were indicated in Fig. 2b–g.

### Antiviral impact of the ZnO/AC nanocomposite against HSV1 virus

**Figure 3:**
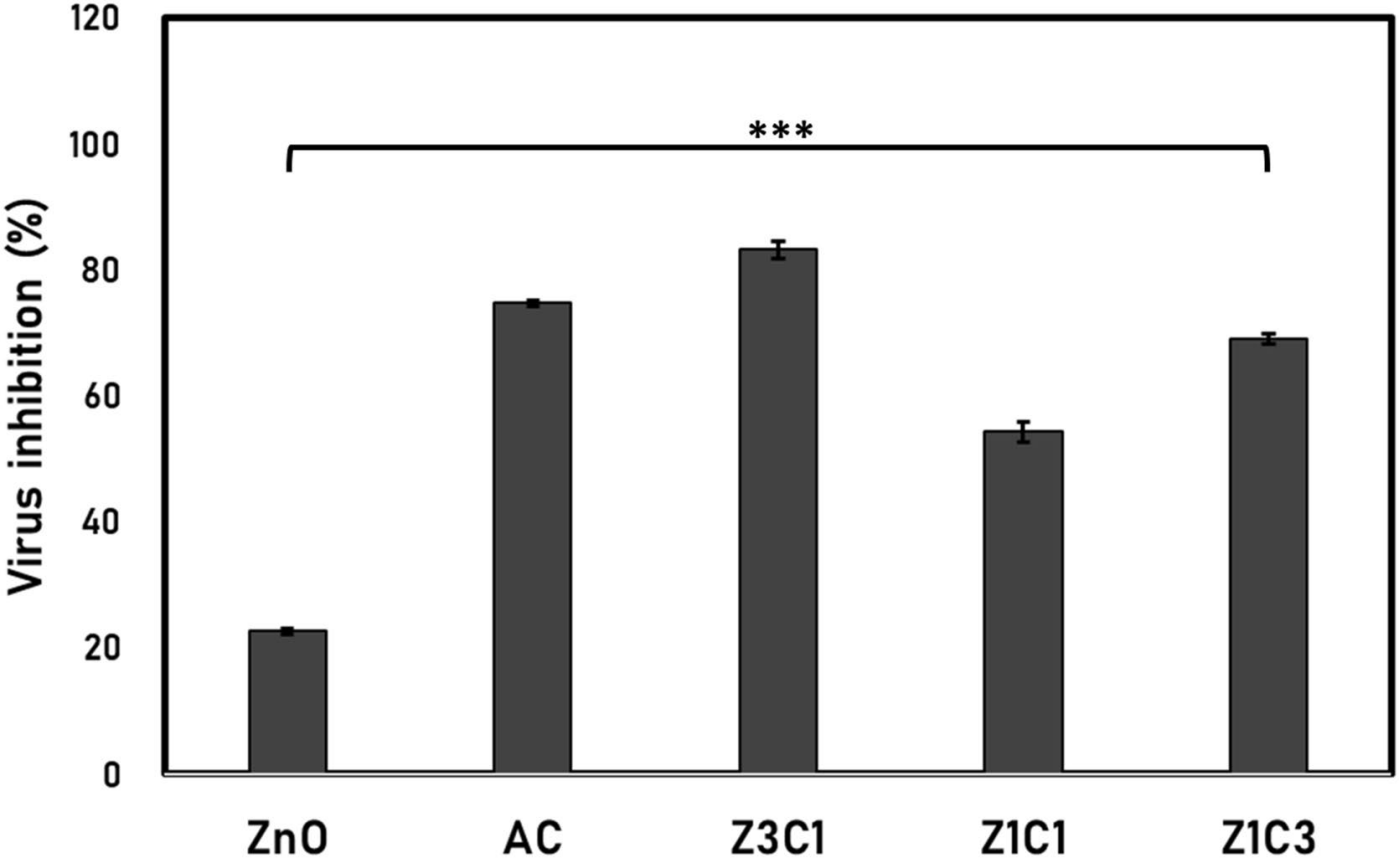
Antiviral impact of the synthesized neat ZnO NPs and AC and ZnO NPs/AC nanocomposites on the activity of (HSV1). The error bars are the estimated standard deviations (n = 3), ***p < 0.001.

Regarding the antiviral influence of the synthesized ZnO/AC nanocomposites against *Herpes simplex* (HSV1) virus, all the samples exerted variant antiviral impact. The formula (Z3C1) exhibited the apical impairing impact against the virus HSV1 by its minimal non-toxic concentration (MNTC) of 1 mg/mL, reaching more than 83% inhibition compared to the control culture (Fig. 3). Interestingly, the antiviral influence of the formula (Z3C1) exceeded the impact of the neat ZnO NPs and the neat AC if measured separately. It is considered another significant proof that the synthesis route succeeded in creating a synergistic combination between the two components of ZnO NPs and AC.

The distinctive antiviral impact of the ZnO NPs was widely studied^21–23^. It was assumed that the selected compositing route is efficient enough to minimize clogging of the generated nanoparticles and to produce nanoparticles with an optimized size as well, where the tested nanoparticles had the potentiality to diffuse into the cells and interfere with the viral replication process. Another factor to be highlighted is the efficacy of the AC particles that may neutralize the viral activity by trapping the viral units inside their structural nano-sized pores.

These expectations were approved previously through Yadavalli et al.^24^, as they attained about 40–60% infection decrease for the HSV-1 and HSV-2 using 0.8 and 1 mg/mL of the highly porous activated carbon (HPAC) solely. They hypothesized that these outstanding results are mainly related to the tremendous potentiality of the utilized activated carbon (AC) to trap viral units inside its nano-pores, preventing them from entering and infecting the cells. Furthermore, the study explained also how the AC exerted extensive support for the efficacy of the combining common antiviral agent, the acyclovir (ACV), where its antiviral impact was remarkably enhanced upon blending with AC.

As it was proved that the sample (Z3C1), that was prepared through mixing ratio ZnO NPs:AC of 3:1, attained the highest efficiency against both the bacterial strain *S. aureus* and virus HSV1. Accordingly, the main chemical and physical properties of this superior ZnO NPs/AC nanocomposite have been investigated adopting various techniques.

### Characterization of ZnO NPs/AC nanocomposite with 3:1 weight ratio

**Figure 4:**
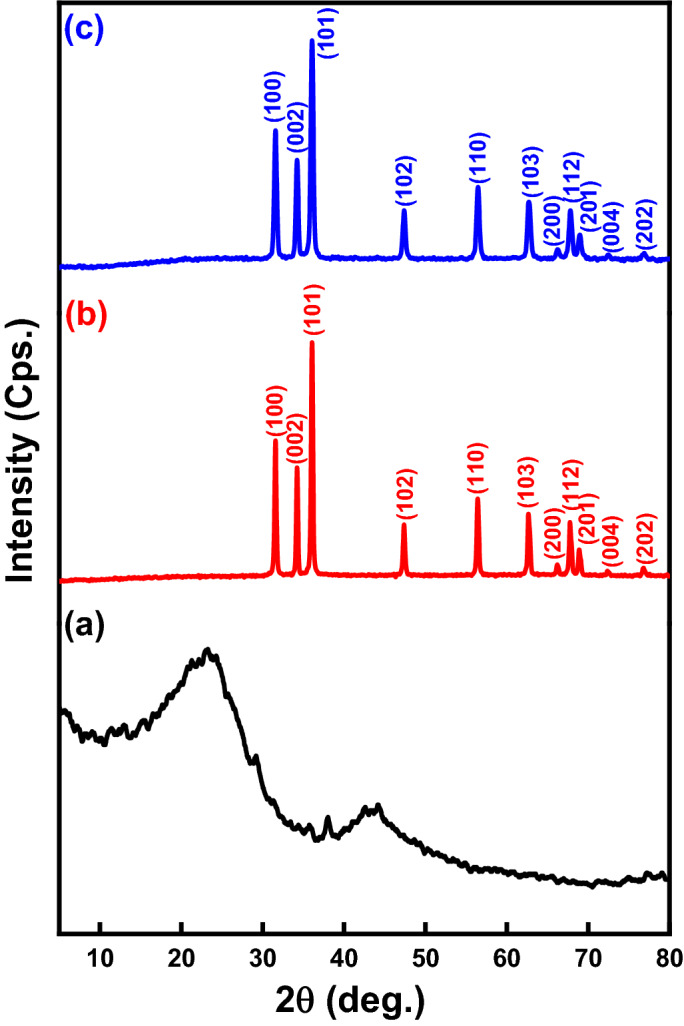
XRD patterns of (**a**) AC, (**b**) ZnO NPs, and (**c**) (Z3C1) nanocomposite.

The crystallinity degree of AC, ZnO NPs, and (Z3C1) nanocomposite with a 3:1 weight ratio was compared as shown in Fig. 4. The XRD patterns of the sample (Z3C1) demonstrated that the all-characteristic peaks of wurtzite hexagonal ZnO NPs according to the JCPDS (36–1451) were indexed at 31.74°, 34.39°, 36.24°, 47.58°, 56.58°, 62.87°, 66.31°, 67.89°, 69.07°, 72.45° and 76.86° with preeminenet crystallinity and with no impurities^17,25^. The d-spacing (Ǻ) of the most significant hkℓ planes (100, 002, and 101) were 2.83335, 2.61952, and 2.49014, respectively. The broad peak with a lower crystallinity degree from 21°-22° is attributed to the diffraction planes of AC^13,26^. Despite the amorphous nature of AC, the ZnO NPs crystallinity was predominated in XRD patterns due to the low content of AC in the fabricated nanocomposite^26^.

**Figure 5:**
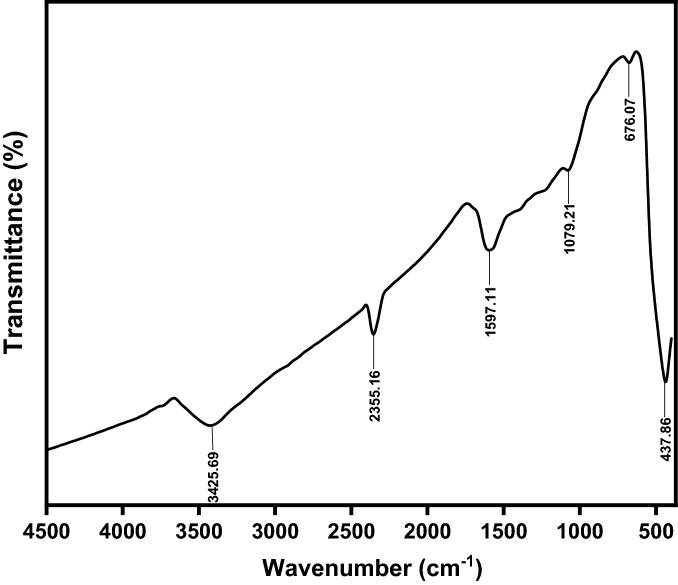
FTIR spectra of the synthesized (Z3C1) nanocomposite.

FTIR spectrum of the sample (Z3C1) nanocomposite was shown in Fig. 5. The bands at 437 and 676 cm^−1^ are attributed to the stretching vibration of the Zn–O bond in ZnO NPs/AC^17,27^. FTIR spectrum shows broadband at 3425 cm^−1^ indicating the stretching vibration of the hydroxyl group of the adsorbed water. On contrary, the stretching vibrations of the C=O bond were observed at 1079, 1597, and 2355 cm^−1^^28^. The aforementioned FTIR data confirmed the excellent incorporation of the different components of the nanocomposite.

**Figure 6:**
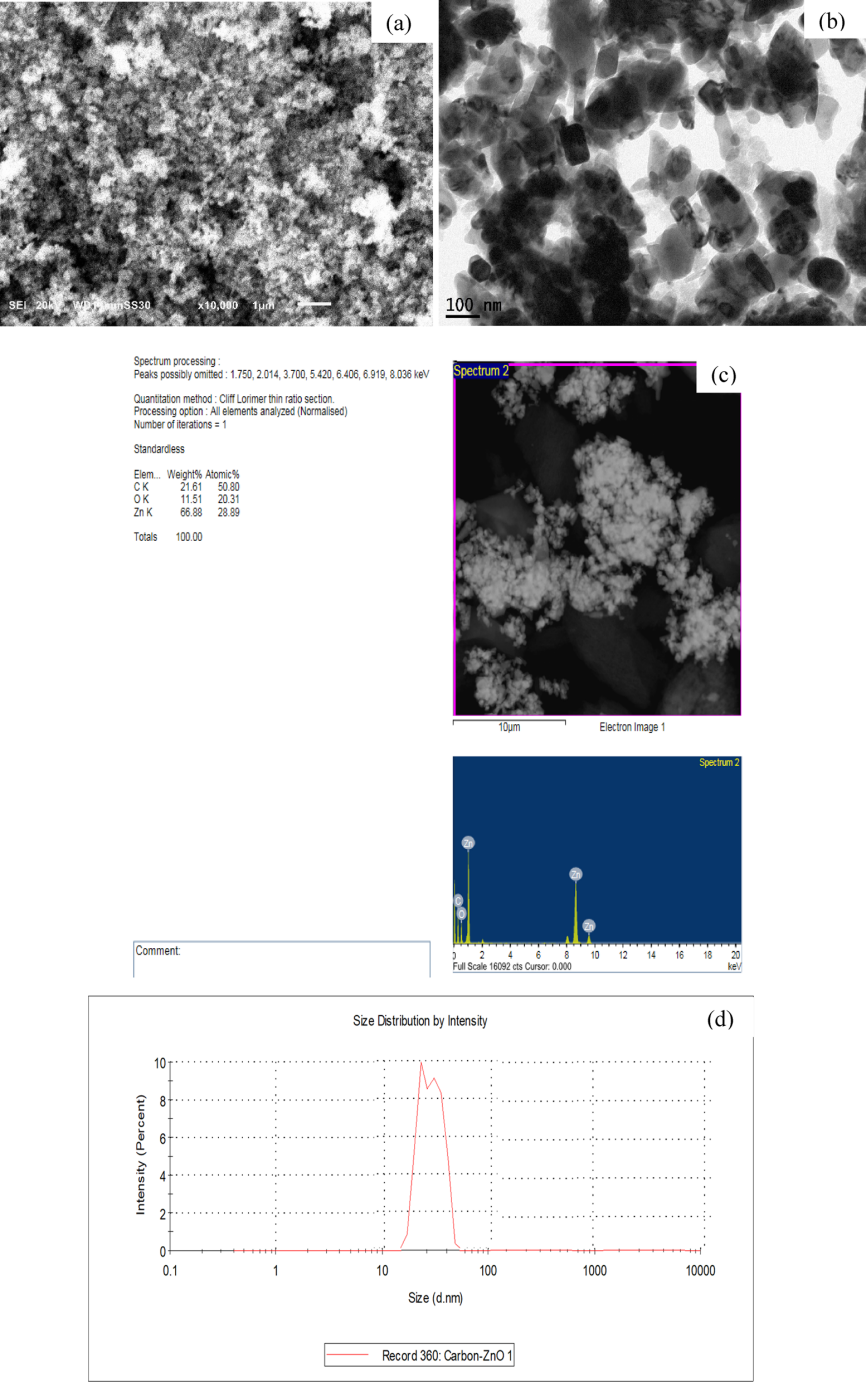
(**a**) SEM, (**b**) TEM, (**c**) EDX, and (**d**) size distribution images of the synthesized (Z3C1) nanocomposite.

Figure 6a,b,c, and d shows the SEM, TEM, EDX, and size distribution images of (Z3C1) nanocomposite, respectively. The SEM, TEM, and size distribution images illustrated the spherical shape of the nanocomposite with particles size swung from 30 to 70 nm. The observant agglomeration in the fabricated composite investigates the successful incorporation between AC and ZnO NPs. Moreover, it was observed that the majority of particles are spherical. Where the bright particles of ZnO NPs were covered with the amorphous darkness AC particles. On the other hand, the elemental mapping (Fig. 6c) investigated the existence of C, Zn, and O in the fabricated nanocomposite. Furthermore, the EDX pattern of the sample (Z3C1) nanocomposite reaffirmed the presence of the aforementioned elements.

**Figure 7:**
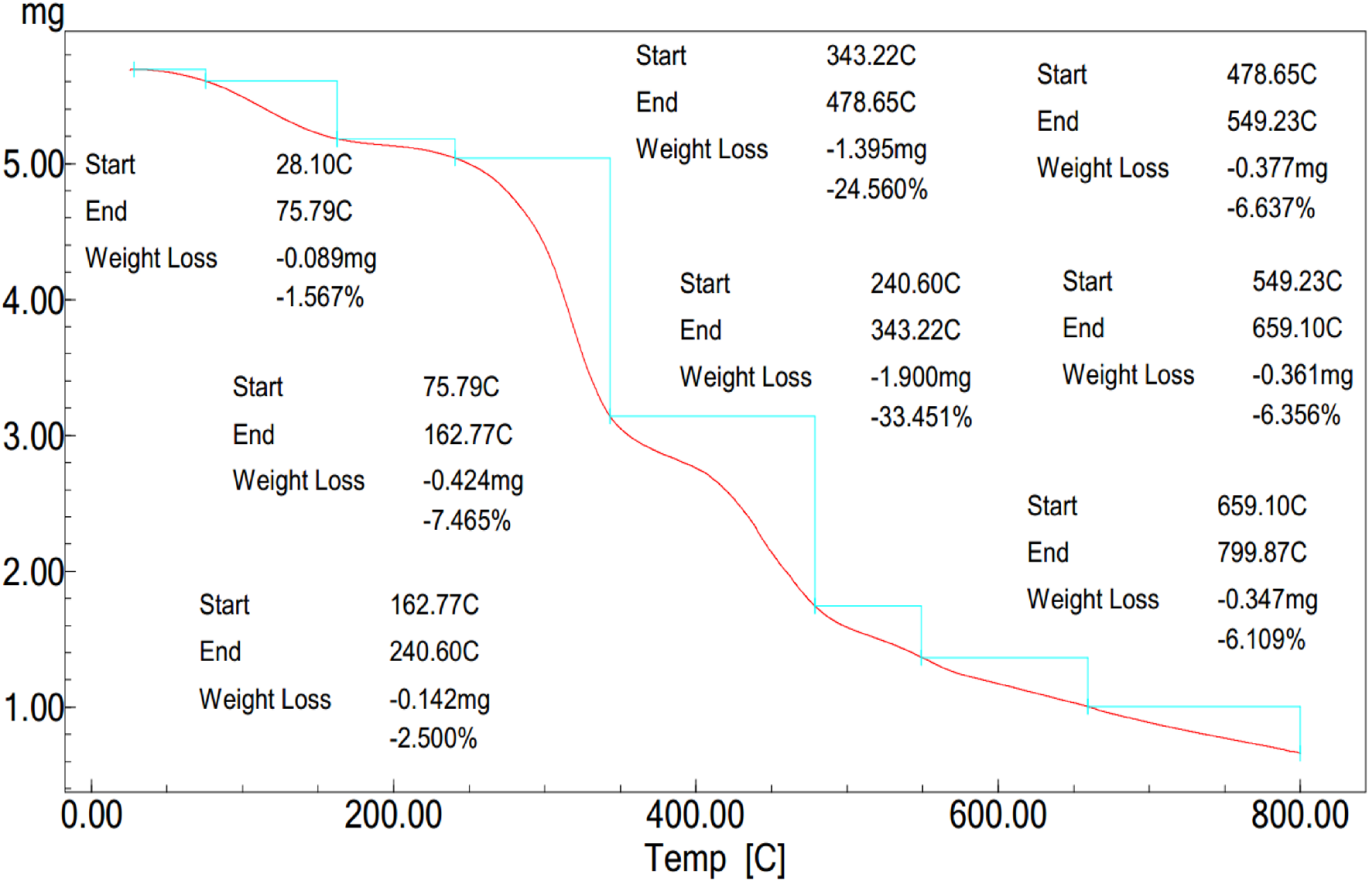
TGA curve of the fabricated (Z3C1) nanocomposite.

Thermogravimetric analysis of the formula (Z3C1) nanocomposite was depicted in Fig. 7, where many weight-loss steps were observed. The first weight-loss stage (∼8%) up to 163 °C that can be attributed to the loss of water molecules and atmospheric gases that are incorporated with the prepared fabricated (Z3C1) nanocomposite^13,29^. The next weight loss step (∼58%) swung from 240 to 480 °C which was assigned to the cleavage of the backbone of the fabricated biocomposite^13^. From the aforementioned data, the fabricated (Z3C1) nanocomposite possesses good thermal stability compared with other bio nanomaterials^13,29^.

**Figure 8:**
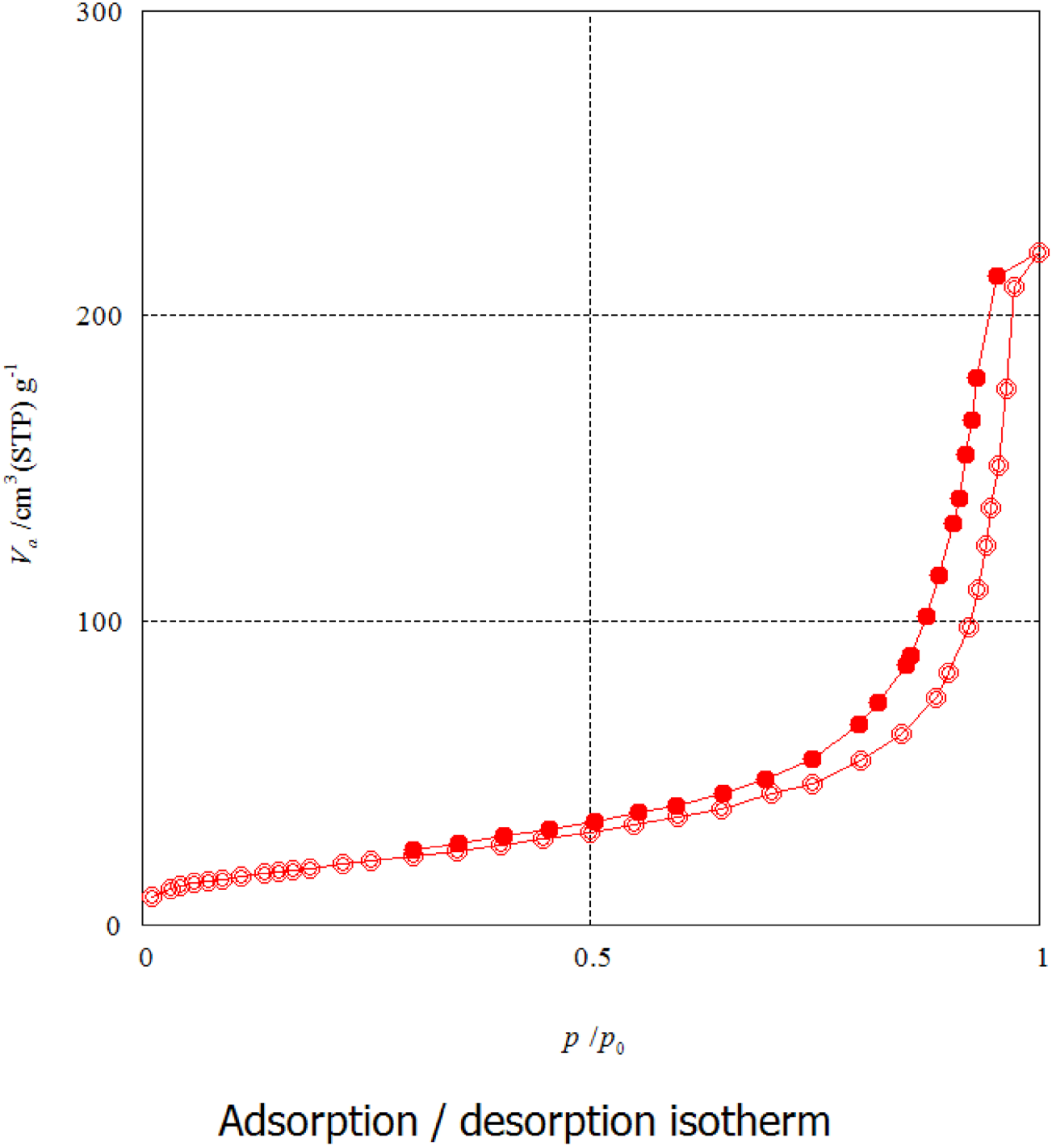
N_2_ adsorption/desorption isotherm of the prepared (Z3C1) nanocomposite.

The specific surface area of the formula (Z3C1) recorded about 66.27 m^2^/g, as well as the total pore diameter, was measured to be 0.25 cm^3^/g as shown in Fig. 8. The N_2_ adsorption/desorption isotherm was type IV, presenting a broad hysteresis loop in the relative pressure (*P*/*P*_0_) range of 0.5–1.0, which confirms the mesoporosity of the fabricated ZnO NPs/AC nanocomposite^30^. According to these results, the fabricated ZnO/AC nanocomposite exerted significant surface area and porosity, which confirms its bioactivity for trapping and disinfection of both the bacterial strain *S. aureus* and virus HSV^13,29^.

## Conclusion

We fabricated ZnO NPs and AC nanoparticles through green routes, before fabricating three nanocomposites with different mass ratios. The synthesized ZnO NPs/AC nanocomposites in addition to the crude ZnO NPs and AC were examined for their antibacterial, antifungal, and antiviral effects. Furthermore, their cytotoxicity on the human WI38 cell line was evaluated. Out of the examined nanocomposites and nanoparticles; the formula (Z3C1) exhibited the best antimicrobial and antiviral activities. As it was recorded, the formula (Z3C1) impaired about 83% of the HSV1 count by its minimal non-toxic concentration (MNTC) of 1 mg/mL. These distinctive antimicrobials and antiviral activities are owed to the immense combination of the ZnO NPs and AC, the elevated surface area, and the nano scale nature of the fabricated ZnO/AC composite. The elaborated incorporation of ZnO NPs onto AC was further proved from the characterization results of both FTIR and XRD of the fabricated ZnO NPs/AC nanocomposite.

## Materials and methods

### Materials

The experimental research on plants, including the collection of plant material, complied with the relevant institutional, national, and international guidelines and legislation. The water hyacinth *(Eichhornia Crassipes)* was collected from Damanhour, Egypt as a raw source for activated carbon. Anhydrous zinc chloride (98%, ZnCl_2_), sodium hydroxide (NaOH), polyvinyl alcohol (PVA) 96.5% (M. Wt. 85,000–124,000 g/mol), and absolute ethanol (C_2_H_5_OH) were procured from Sigma-Aldrich. All chemicals were utilized without any further modifications. WI-38 normal lung fibroblast cells (ATCC® CCL-75™) were purchased from VACSERA CO., Cell Culture Research Unit, Giza, Egypt.

### Synthesis of AC NPs from raw water hyacinth

The raw water hyacinth was washed with distilled water several times to eliminate any foreign materials and dried for two days at 50 °C. Additionally, the dried plants were grinded with a high-speed multifunction grinder (HC-500Y, Yongkang Shengshi Industry and Trade Co., China) and sieved with a 1 mm sieve. To improve the active surface area of the sieved material, the water hyacinth was chemically modified by alkali treatment. Consequently, 50 mL of 1 M NaOH were added to five grams of the 1 mm sieved raw material. The slurry was mixed at an agitation speed of 200 cycles/min for 4 h at 50 °C then filtered and dried at 60 °C. To enhance the adsorption properties of the fabricated activated carbon, 50 mL of 1 M zinc chloride were mixed with 5 g of the prepared chemically activated AC for six hours under continuous stirring. This slurry was filtered and washed several times with distilled water to eliminate any unreacted components. The resulted material was further dried for 24 h at 100 °C. Finally, the produced material was calcined at a muffle furnace at 400 °C for 2 h. The fabricated activated carbon was chilled under nitrogen flow overnight at room temperature^13^.

### Fabrication of ZnO NPs

A volume of 150 mL of 20 mM of ZnCl_2_ in the presence of 25 mg of PVA as stabilizing were prepared^14,15,31^. Afterwards, 50 mL of 5 M NaOH were added dropwise to the aforementioned mixture. Moreover, the resulting solution was maintained for 1 h using a microwave (Thomson-Combi1, USA) at 800 W. The produced powder was washed several times with distilled water and absolute ethanol to remove the unreacted compounds. Finally, the fabricated material was dried at 60 °C for 24 h and weighed.

### Preparation of ZnO NPs/AC nanocomposites

In order to immobilize the ZnO NPs with the synthesised AC, the ball-mill (Photon Scientific, XQM-0.4A) technique was adopted. The nanocomposites were prepared with different immobilization ratios of ZnO NPs:AC (4:0, 0:4, 3:1, 1:1, and 1:3) and named (ZnO, AC, Z3C1, Z1C1 and Z1C3, respectively). All the different selected ratios were inserted in the ball-mill jar with 1:20 for materials: balls ratio for 60 min at 500 rpm to obtain ZnO NPs/AC nanocomposites.

### Antimicrobial activity of the ZnO NPs/AC nanocomposites

All the synthesized ZnO NPs/AC nanocomposites were examined for their antimicrobial activity against six microorganisms through the clear zone technique. These studied microorganisms included two Gram-negative bacteria of *Escherichia coli* (ATCC® 8739™) and *Klebsiella pneumonia* (ATCC® 13,883™); two Gram-positive bacteria of *Bacillus Subtilis* (ATCC® 6633™) and *Staphylococcus aureus* (ATCC® 6538™); and two unicellular fungi of *Candida albicans* (ATCC® 10,231™) and multicellular fungus *Aspergillus niger* (ATCC® 16,404™). The bacterial strains and the *C. albicans* strain were seeded in a nutrient agar medium, while the filamentous fungi *A. niger* was grown on Czapek's medium. The antimicrobial activity of the differently prepared nanocomposites was tested according to the steps mentioned in our previous study^32^. Briefly, the impact of ZnO NPs/AC nanocomposites on the growth of the different tested bacteria isolates were grown in nutrient broth (NB) medium at specific concentrations at 30 °C for 24 h. Subsequently, 1 mL of culture was utilized for serial dilution, then grown on nutrient agar (NA) medium for 24 h at 30 °C before the colony-forming units (CFU) were counted. The findings were compared with a broad-spectrum antimicrobial agent (Gentamicin).

### Determination of cytotoxicity of the synthesized ZnO NPs/AC nanocomposites on the human cell line (WI38)

The influence of the neat ZnO NPs, neat AC, and the synthesized ZnO NPs/AC nanocomposites on the viability of the WI38 cell line was inspected. The samples were sterilized by filtration through Millipore 0.45 μm; then, a cytotoxicity assay was performed by 3-(4, 5-dimethylthiazol-2-yl)-2,5-diphenyltetrazolium bromide (MTT) assay as reported by Felix and his co-workers^33^ with slight modifications.

In brief, WI38 cell lines were grown at a concentration of 1 × 10^5^ cells per well in 96-well microtiter plates for 24 h at 37 °C until developing a monolayer sheet, before decanting the growth medium and washing twice with washing media. A set of five concentrations of each sample (1000, 500, 250, 125, and 62.5 µg/mL) were prepared, where 100 µL of each dilution to different 3 wells leaving 3 wells as control, including only maintenance medium. The plates were incubated at 37 °C. MTT solution was then prepared (5 mg/mL in PBS) (Bio Basic Canada Inc.), then we added 20 µL of MTT solution to each well, then moved to a shaker working at 150 rpm for 5 min, to thoroughly mix the MTT into the media. The plates were incubated for 3 h to allow the MTT to be metabolized. Then, we dumped off the media, and the plates and the residual formazan (MTT metabolic product) were re-suspended in 200 µL DMSO, then placed on a shaking table at 150 rpm for 5 min, to thoroughly mix the formazan into the solvent.

Finally, the optical density at 570 nm for all wells was determined, subtracting the background at 630 nm as a correlating factor of cell viability, where cell viability at each treatment can be concluded from the Eq. 1:

1 $$ {\text{Cell viability }}\left( \% \right) = \frac{{{\text{OD}}_{570 - 630} \left( {treatment} \right)}}{{{\text{OD}}_{570 - 630} \left( {Negative control} \right)}} \times 100\, \left( \% \right) $$

### Antiviral effect of the synthesized ZnO NPs/AC nanocomposites

The neat ZnO NPs, neat AC, and the synthesized ZnO NPs/AC nanocomposites were determined for their significance in the activity of the virus *Herpes simplex* (HSV1). First, the MTT test was accomplished versus Vero cell lines (ATCC® CCL-81™- *Cercopithecus aethiops* kidney normal) to determine the minimal non-toxic concentration (MNTC) through the MTT assay method that was detailed in the previous section.

Afterward, we added 1 × 10^4^ cells in 200 µL media per well in a 96 well plate, and 3 wells were left empty for blank controls. The plates were incubated overnight to allow the cells to attach to the wells. The equal volume (1:1 v/v) of the non-fatal dilutions of each ZnO NPs/AC nanocomposite sample and the virus suspension was incubated for 1 h. Then, 100 µL of the viral/sample suspension was added to the relevant well. The plates were gently shaken and then incubated for 24 h to allow the virus to become operative.

Eventually, 20 µL of MTT/PBS solution was added to each well and following the same steps mentioned in the previous section until reading the optical density at 570 nm and concluding the viral activity.

### Characterization of the ZnO NPs/AC nanocomposites with a 3:1 weight ratio

The selected ZnO NPs/AC nanocomposite material with a weight ratio of 3:1 was characterized by X-ray diffractometer (XRD) (Schimadzu-7000, Shimadzu Corporation, Kyoto, Japan), Fourier transform infrared (FTIR) (Bruker, Bremen, Germany), Scanning electron microscope (SEM) (JEOL JSM-6010LV, Japan), transmission electron microscope (TEM) (JEOL, JEM-2100, Japan with an accelerating voltage of 80 kV), and thermogravimetric analysis (TGA) (Shimadzu TGA-50 instrument). Furthermore, the Brunauer–Emmett–Teller (BET) surface area was determined using a surface area analyser (Beckman Coulter SA3100, Brea, CA, USA) by measurement of the N_2_ adsorption–desorption isotherms at 77 K. All samples were degassed before measurements under vacuum at room temperature for 12 h.

## Data Availability

All the data of this study are included in the submitted manuscript. For further data, please contact the corresponding author (H.S.) via hassan.shokry@gmail.com.
